# Symptomatic Aortic Valve Mass - Cardiac Work-Up Challenges and Role of Computed Tomography Angiography: A Case Report

**DOI:** 10.21470/1678-9741-2018-0151

**Published:** 2019

**Authors:** Victor S. Reis, Darren C. Tsang, Donald B. Williams, Roger G. Carrillo

**Affiliations:** 1 Division of Cardiothoracic Surgery, University of Miami Miller School of Medicine, Miami, Florida.

**Keywords:** Heart Neoplasms - Complications, Angina Pectoris - Etiology, X-Ray Computed Tomography, Magnetic Resonance Imaging

## Abstract

Cardiac papillary fibroelastoma are rare, benign cardiac tumors that may lead to lethal complications from embolization or valvular dysfunction if left untreated. When working up symptomatic tumors with concomitant angina, traditional diagnostic studies such as cardiac catheterization may predispose the patient to embolic complications if the mass is located in the path of the catheter. Newer, non-invasive diagnostic testing, such as cardiac magnetic resonance imaging or dynamic computed tomography angiography, may be considered in lieu of invasive approaches to avoid potentially devastating complications. We herein present a case report of a 77-year-old female with a symptomatic aortic valve tumor and describe our diagnostic strategy and management.

**Table:** 

Abbreviations, acronyms & symbols	
CPF	= Cardiac papillary fibroelastomas
CT	= Computed tomography
MRI	= Magnetic resonance imaging

## INTRODUCTION

Primary cardiac tumors are rare entities that occur between 0.001%-0.3%, based on autopsy reports^[1]^. Cardiac valve tumors such as cardiac papillary fibroelastomas (CPF) are a subset of masses that, if left untreated, can lead to devastating complications including embolization, stroke, and death^[2]^. When a cardiac mass is suspected, a thorough evaluation of the patient’s cardiac anatomy and function using echocardiography is imperative. When angina is present, additional diagnostic testing should be performed to determine whether symptoms originate from the mass or another cardiac pathology. Unfortunately, invasive testing such as cardiac catheterization may predispose these patients to embolic complications. We propose that non-invasive coronary angiography using magnetic resonance imaging (MRI) or dynamic computed tomography (CT) may be considered in lieu of coronary catheterization in this uncommon patient population. We describe a patient with a symptomatic aortic valve tumor that was successfully diagnosed with non-invasive angiography and managed with valve-sparing excision. Informed consent and Institutional Review Board approval were obtained for reporting this case.

## CASE REPORT

### Patient Information

A 77-year-old female with a history of myocardial ischemia and coronary artery disease who underwent percutaneous coronary intervention nine years ago had recently developed exertional angina, shortness of breath, and near syncope. Her symptoms began a few months prior but had recently become more severe.

### Clinical Findings

On auscultation, she had a systolic ejection murmur that radiated to the neck, bilaterally. The remainder of her physical exam was otherwise unremarkable.

### Diagnostic Assessment

**Fig. 1:**
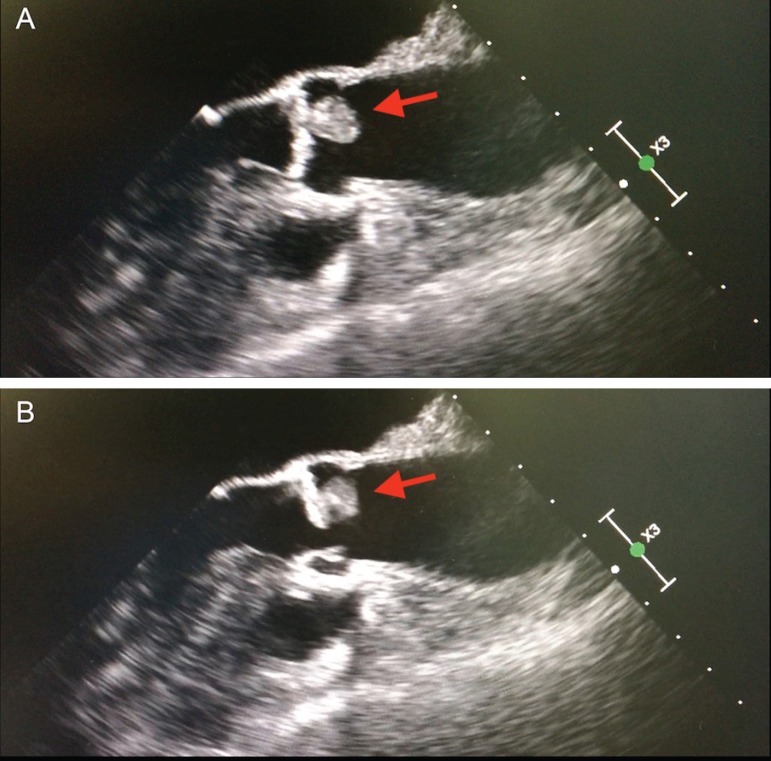
Transesophageal echocardiography A) 1.5 x 0.8 cm mass attached to the left coronary leaflet, B) mass preventing the leaflet from opening completely.

**Fig. 2:**
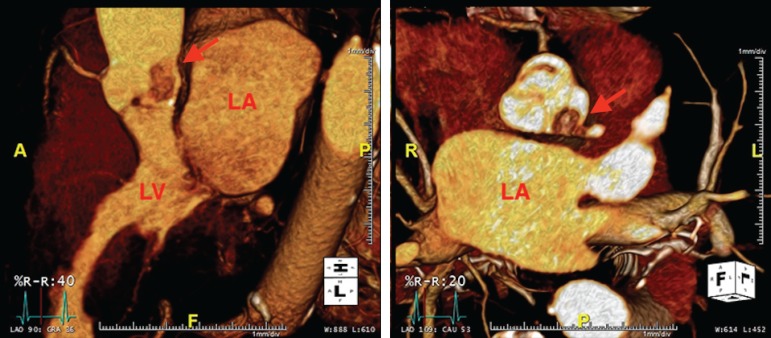
3-D CT revealing mass abutting the ostia of the left main coronary artery in systolic phase.

**Fig. 3:**
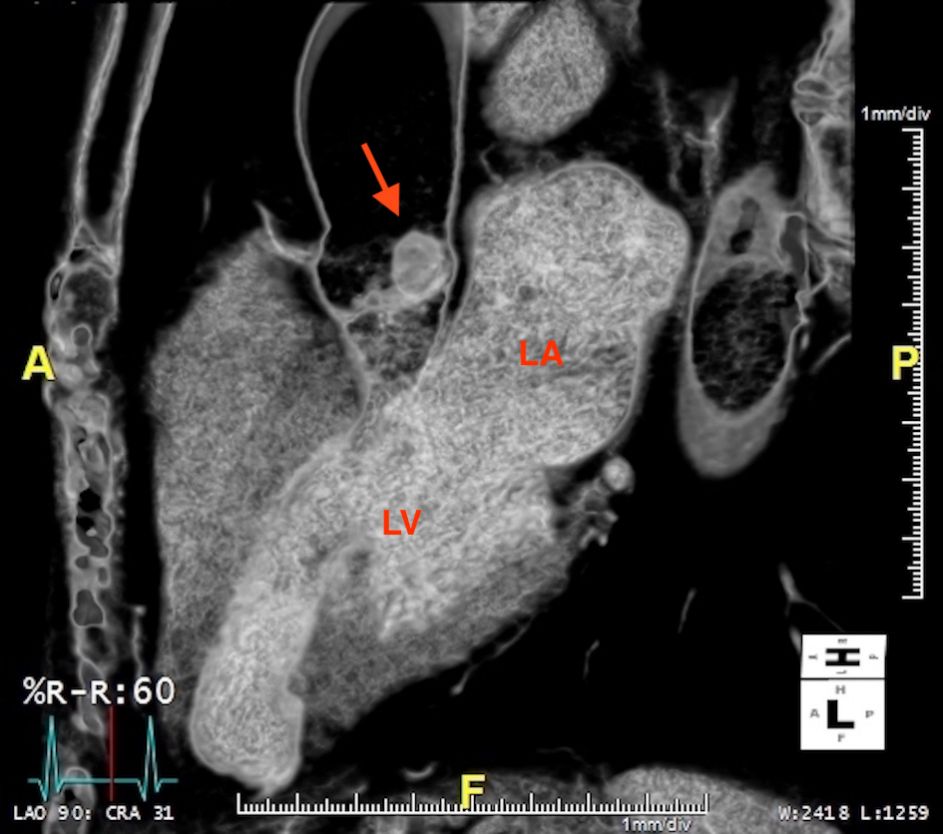
3-D CT of the chest in diastolic phase .

Two-dimensional echocardiogram showed a 1.5 cm x 0.8 cm globular mass on the wall of the left sinus of Valsalva just above the aortic valve (Figure 1) and moderately reduced aortic valve area of 1.5 cm^2^ as estimated by the continuity equation. Her mean aortic valve gradient was 10 mmHg and her peak aortic velocity was 2.2 m/s. Left ventricular ejection fraction was 60% and no mitral regurgitation was detected. Given her history of coronary artery disease, she required an evaluation of her coronary arteries to rule out significant atherosclerosis. However, the location of the mass on the left coronary cusp would have made it precarious to cannulate the coronary ostium for catheterization. Thus, we elected to perform noninvasive evaluation using dynamic CT angiography. The study did not show evidence of obstructive coronary artery disease but did prominently display the mass during the cardiac cycle (Figure 2 and 3). It appeared the mass would abut the left coronary ostium during systole, which may have explained her symptoms.

### Therapeutic Intervention

**Fig. 4:**
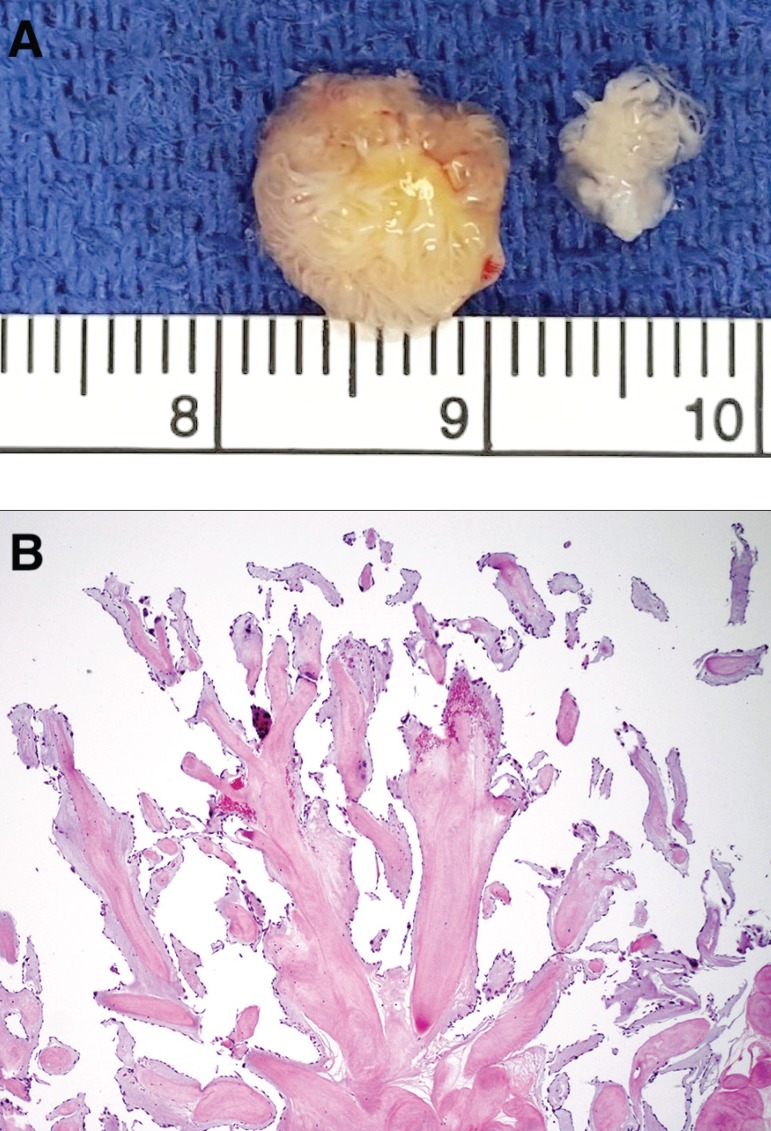
A) Gross specimen measuring 1.5 cm in diameter with characteristic frond-like appearance B) Hematoxylin and eosin stain revealing avascular, finger-like processes containing elastic tissue and dense collagen covered by a single layer of endocardial cells.

The mass was removed surgically through a median sternotomy. The patient was placed on cardiopulmonary bypass and the heart was arrested. The aorta was opened, and upon inspection of the aortic valve, the mass was found to originate from a stalk on the left coronary cusp. The 1.5 cm mass was found to have a sessile attachment to the free edge of the left coronary leaflet and was removed without disrupting the architecture of the aortic valve (Figure 4A). Post-operative echocardiography showed no aortic insufficiency and improvement in aortic valve function. The aortic valve area had increased to 1.7cm^2^ while her mean aortic valve pressure gradient and peak aortic velocity had decreased to 6 mmHg and 1.6 m/s, respectively.

### Follow-up/Outcomes

Her post-operative course was unremarkable. She was extubated a few hours after surgery, her chest tubes were removed post-op day 2 and she was transferred to the floor post-op day 3. From postoperative days 4 to 8, the patient was medically stable but awaited social placement. She was evaluated by physical therapy for deconditioning and was recommended outpatient rehabilitation, given supplemental oxygen, and discharged on postoperative day 8. Pathology revealed a cardiac papillary fibroelastoma (CPF).

## DISCUSSION

CPFs are rare, benign lesions that represent 10% of all primary cardiac tumors and present frequently on the valves, with a predilection for the aortic valve^[1,3]^. Histological examination reveals that embolic complications often occur from either the fragile papillary fronds of the tumor or thrombus aggregation on the superficial endothelial layer (Figure 4B) ^[2,4]^. In several retrospective single-center studies, roughly 32% of patients were symptomatic and presented with neurological events (syncope, transient ischemic attack, stroke), coronary ischemia (angina, myocardial infarction, cardiac arrest, sudden death), peripheral embolization, outflow obstruction, or valvular insufficiency^[2-4]^. Our patient’s presentation was more consistent with aortic stenosis. Her tumor was located on the free edge of the left coronary leaflet on the aortic side. At 1.5 cm, this tumor may have prevented the left leaflet from opening completely, resulting in left ventricular outflow obstruction. In addition, the tumor may have transiently occluded the orifice to the left coronary artery, leading to chest discomfort and dyspnea on exertion. Upon suspicion of a CPF, the friability, mobility, and location of the mass should be evaluated in order to decide on the appropriate diagnosis and treatment. Clinical history may indicate tumor friability, and echocardiogram may reveal tumor mobility and location^[4]^. However, given our patient’s symptoms and history of coronary artery disease, it was also imperative to determine the source of her angina and consider the need for coronary artery bypass. Due to the location of the mass, it was felt that conventional catheter angiography was contraindicated owing to the risk of systemic embolization. Dynamic CT angiography was a valuable alternative that provided useful information regarding coronary blood flow and helped rule out hemodynamically significant stenosis; it provided higher-quality, three-dimensional imaging that allowed us to delineate her coronary anatomy, characterize the mass, and complete her cardiac workup prior to surgery. The additional radiation exposure and risk of contrast-induced nephropathy were warranted. Without appropriate assessment, undiagnosed coronary artery disease may go untreated at the time of surgery, increasing the risk of postoperative complications such as myocardial infarction and death.

Review of the literature revealed that echocardiography was the modality most often used to diagnosis CPFs. In a single-center retrospective review of 162 pathologically confirmed cases, Sun et al.^[3]^ reported the sensitivity and specificity of transthoracic echocardiography in the detection of CPF ≥0.2 cm to be 88.9% and 87.8%, respectively. Echocardiography may provide a dynamic view of the mass and, if associated with a valve, may describe valvular pathology attributed to the mass’s presence. However, recent advancements in spatial and temporal resolution allow dynamic CT and cardiac magnetic resonance imaging to play a more prominent role in the workup of cardiac masses. Carpenter et al. report a case of exertional angina that necessitated the stepwise use of stress electrocardiography, catheter angiography, dynamic CT angiography, and cardiac MRI to diagnose a CPF^[5]^. Additionally, our case further highlights the utility of these newer modalities in mitigating the risk of embolic complications. In cases of suspected concomitant ischemic pathology, we propose that non-invasive coronary angiography using MRI or dynamic CT may be considered in lieu of coronary catheterization in this uncommon patient population. Surgical treatment is indicated for symptomatic patients to prevent further ischemic or thromboembolic events. Excision is curative with valve-sparing procedures being feasible in the majority of cases^[2,4]^. However, the surgical management of asymptomatic patients with an incidental cardiac valve tumor is controversial. Gowda et al. reported 12 tumor-related deaths from embolization or obstruction of the coronaries in the cases of 25 medically-treated CPF patients, suggesting that the likelihood of developing significant symptoms over time should not be ignored^[2]^. Thus, Ikegami et al., in their review, recommend surgical treatment for all incidentally found CPF^[4]^. Furthermore, Miller et al. suggest patients be placed on systemic anticoagulation until the tumor can be removed^[6]^. Since the incidence of cardiac valve tumors is rare, it is unlikely that the best management of asymptomatic patients will be clearly elucidated by a randomized control trial. Therefore, we believe that any patient who is otherwise a candidate for cardiac surgery should have left-sided cardiac valve tumors removed, regardless of symptomatology. Right-sided lesions, in the absence of a septal defect, may be managed initially with anticoagulation and need for surgery should be determined on a case-by-case basis.

## CONCLUSION

In conclusion, cardiac valve tumors are extremely rare, with potential for devastating complications. If diagnosed, cardiac catheterization should be avoided to minimize the risk of embolic events. In symptomatic patients, dynamic CT angiography can clarify coronary artery anatomy and define the tumor prior to surgical excision.

**Table:** 

Author's roles & responsibilities	
VSR	Substantial contributions to the conception or design of the work; acquisition; drafting, revising; agreement to be accountable; final approval of the version to be published
DCT	Substantial contributions to the conception or design of the work; drafting; revising; final approval of the version to be published
DBW	Substantial contributions to the acquisition; interpretation; final approval of the version to be published
RGC	Substantial contributions to the conception or design of the work; acquisition; interpretation; drafting; agreement to be accountable; final approval of the version to be published

## References

[1] Anavekar NS, Bonnichsen CR, Foley TA, Morris MF, Martinez MW, Williamson EE (2010). Computed tomography of cardiac pseudotumors and
neoplasms. Radiol Clin North Am.

[2] Gowda RM, Khan IA, Nair CK, Mehta NJ, Vasavada BC, Sacchi TJ (2003). Cardiac papillary fibroelastoma: a comprehensive analysis of 725
cases. Am Heart J.

[3] Sun JP, Asher CR, Yang XS, Cheng GG, Scalia GM, Massed AG (2001). Clinical and echocardiographic characteristics of papillary
fibroelastoma: a retrospective and prospective study in 162
patients. Circulation.

[4] Ikegami H, Andrei AC, Li Z, McCarthy PM, Malaisrie SC (2015). Papillary fibroelastoma of the aortic valve: analysis of 21
cases, including a presentation with cardiac arrest. Tex Heart Inst J.

[5] Carpenter JP, Price S, Rubens MB, Sheppard MN, Moat NE, Morgan A (2011). Aortic papillary fibroelastoma as an unusual cause of angina:
insights from multimodality imaging. Circ Cardiovasc Imaging.

[6] Miller A, Perez A, Pabba S, Shetty V (2017). Aortic valve papillary fibroelastoma causing embolic strokes: a
case report and review. Int Med Case Rep J.

